# Supplementary material for: Tcf1 and Lef1 provide constant supervision to mature CD8+ T cell identity and function by organizing genomic architecture
Source: Nat Commun. 2021 Oct 6;12:5863. doi: 10.1038/s41467-021-26159-1 (PMC8494933; doi:10.1038/s41467-021-26159-1)
Supplement: Supplementary file 1 — Supplementary Information [file 41467_2021_26159_MOESM1_ESM.pdf]

**Shan and Li et al. Supplementary Figure 1**

a

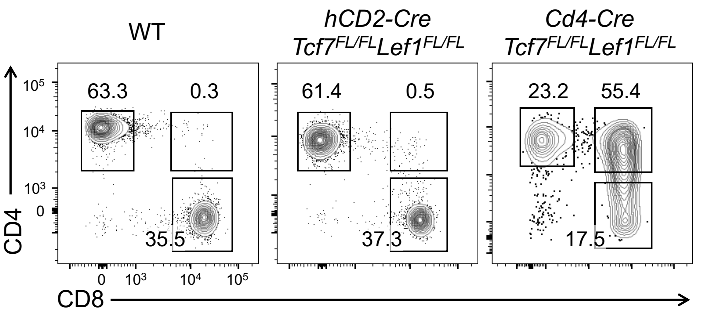


b

| Sample name | Replicates | No. of valid read pairs before deduplication | No. of valid read pairs after deduplication |
|---|---|---|---|
| WT CD8+ | Rep1 | 404,008,692 | 316,712,283 |
| | Rep2 | 561,051,531 | 246,556,142 |
| dKO CD8+ | Rep1 | 418,715,353 | 337,155,850 |
| | Rep2 | 639,231,453 | 123,591,327 |

c

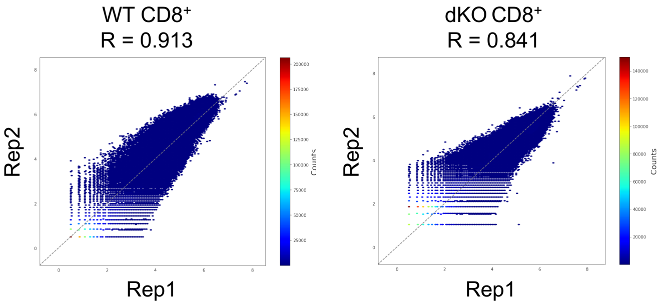


**Supplementary Figure 1. Specific ablation of Tcf1 and Lef1 in mature CD8$^+$ T cells and Hi-C library statistics**.

**a.** Developmental stage-specific deletion of Tcf1 and Lef1 has distinct impact on mature CD8$^+$ T cells. *Tcf7*$^{FL/FL}$*Lef1*$^{FL/FL}$ strain was crossed with *Cd4-Cre* or *hCD2-Cre*, which deleted Tcf1 and Lef1 at the TCRβ$^+$DP thymocytes or mature CD8$^+$ T cells, respectively. Splenic TCRβ$^+$ cells were analyzed for CD4$^+$ and CD8$^+$ T cell lineage distribution. Whereas *Cd4-Cre*-mediated Tcf1/Lef1 deletion resulted in derepression of CD4 coreceptor in CD8$^+$ T cells (right panel), *hCD2-Cre*-mediated deletion did not (middle panel).

**b**. Summary statistics of the Hi-C libraries.

**c**. Scatterplots showing reproducibility of two biological replicates of WT and dKO CD8$^+$ T cell Hi-C libraries. The *x*- and *y*-axis values for each data point (marked with a dot) represent the normalized paired-end tag (PET) counts in replicate 1 (Rep1) and replicate 1 (Rep 2), respectively. The dot color denotes local density. The R values denote Pearson correlation coefficient.

**Shan and Li et al. Supplementary Figure 2**

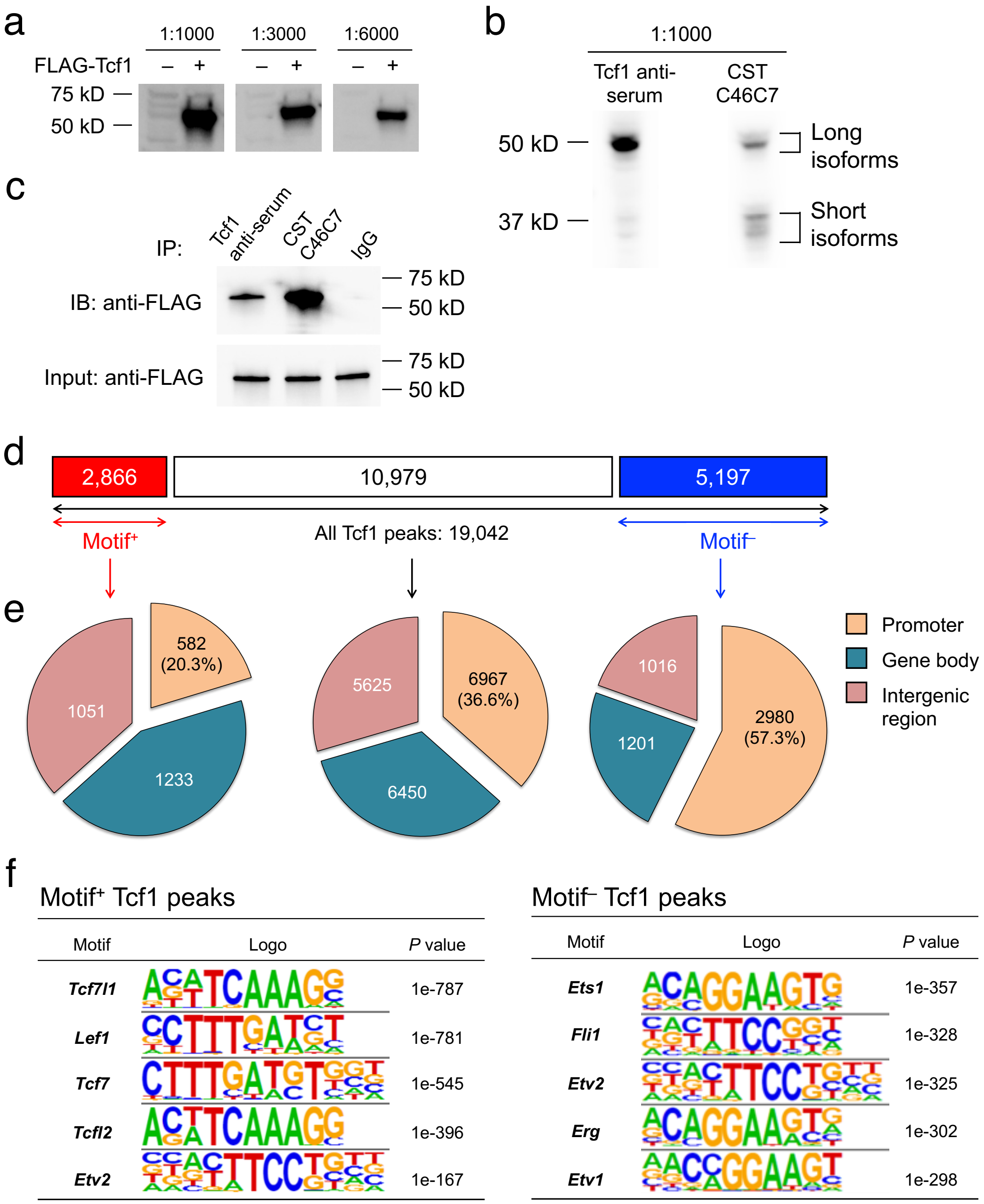


**Supplementary Figure 2. Tcf1 binding peaks exhibit varied degrees of Tcf/Lef motif enrichment.**

**a–c.** Characterization of Tcf1 antiserum generated in house. **a**) FLAG-tagged Tcf1-expressing vector was transfected into 293T cells, and the cell lysates were immunoblotted with the antiserum at various dilutions. *Note that molecular weight of FLAG-tagged Tcf1 was higher than the WT full-length Tcf1 protein at the 45 kDa*. **b**) Cell lysates from sorted naïve $CD8^+$ T cells were immunoblotted with the Tcf1 antiserum or the commercially available Tcf1 antibody (clone C46C7 from Cell Signaling Technologies, CST) at the same dilution. Tcf1 long and short isoforms are marked with brackets. **c**) Cell lysates from 293T cells transfected with FLAG-Tcf1 were immunoprecipitated with the Tcf1

antiserum (4 μl), the CST C46C7 Tcf1 antibody (4 μl, 0.65 μg), or IgG followed by immunoblotting with anti-FLAG antibody. Data are representative from ≥ 2 independent experiments.

**d.** Distinction of Tcf1 binding peaks based on enrichment of Tcf/Lef motifs. High confidence Tcf1 binding peaks were mapped with ChIP-seq in WT $CD8^+$ T cells and analyzed for enrichment of Tcf/Lef motifs using HOMER. A total of 2,866 Tcf1 peaks were strongly enriched for the Tcf/Lef motifs with enrichment score above 7, and hence defined as $Motif^+$ Tcf1 peaks, while 5,197 Tcf1 peaks with enrichment score under 3 were defined as $Motif^–$ Tcf1 peaks.

**e**. Pie charts showing the genomic distribution of all (middle), Motif+ (left), and Motif– (right) Tcf1 peaks. The total numbers of Tcf1 peaks in each category are shown, with the percentages of promoter distribution marked in parentheses.

**f.** *De novo* motif analysis of $Motif^+$ and $Motif^–$ Tcf1 peaks using HOMER. Top five most enriched motifs are listed together with motif logo, and statistical significance is an output from HOMER. Note that *Tcf7*, *Lef1*, *Tcf7l1* and *Tcf7l2* genes encode Tcf1, Lef1, Tcf3 and Tcf4 proteins in the Tcf/Lef family TFs, respectively.

**Shan and Li et al. Supplementary Figure 3**

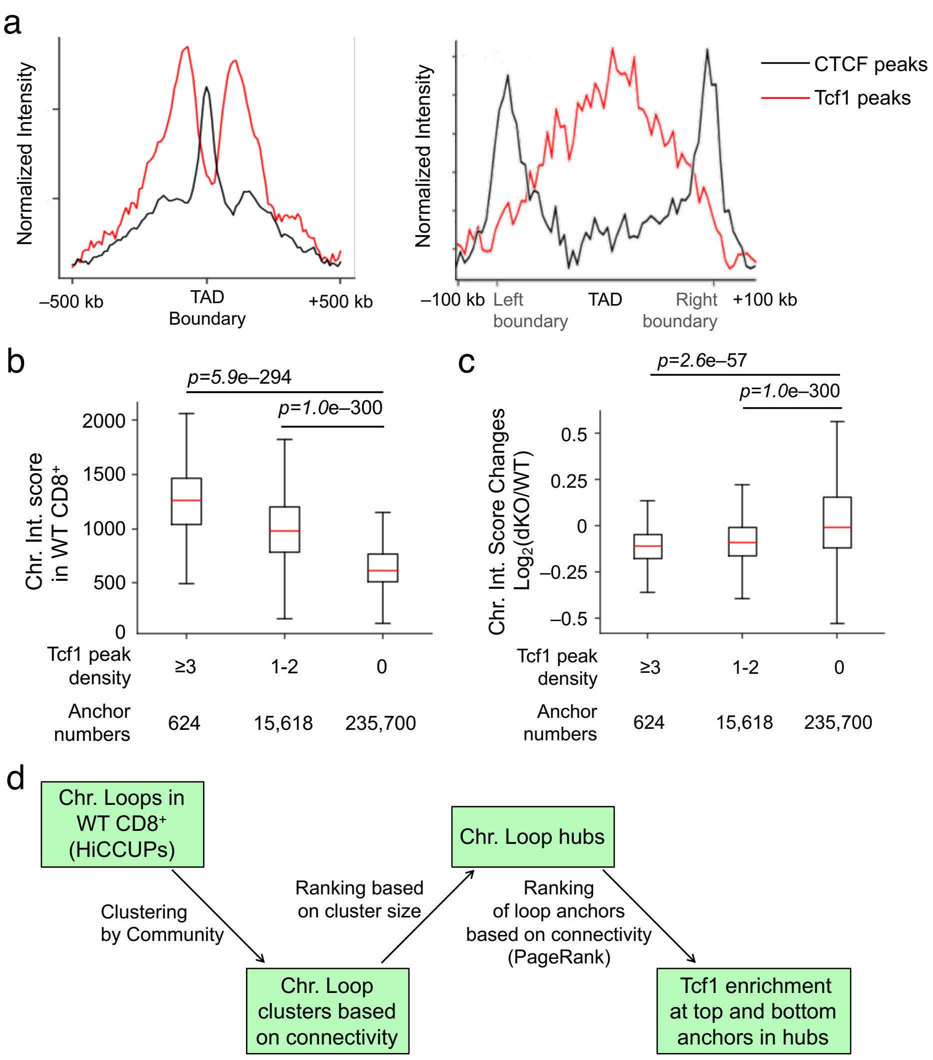


**Supplementary Figure 3. Tcf1 is distributed within TADs and modulates chromatin interactions**.

**a**. CTCF but not Tcf1 is associated with TAD boundaries. TADs were identified in WT CD8$^+$ T cells with the Arrowhead algorithm. In the left panel, the TAD boundary was centered; in the right panel, the TAD was centered with its boundaries aligned in flanking regions. Both panels display the normalized intensity of CTCF or Tcf1 ChIP-seq signals.

**b**. Tcf1 peaks are enriched in chromatin interaction regions. Chromatin interaction (Chr. Int.) anchors were allocated into different groups based on Tcf1 peak numbers in WT CD8$^+$ T cells, and the Chr. Int. score was determined for each anchor.

**c**. Tcf1/Lef1 deficiency diminishes chromatin interactions. Chr. Int. score changes in dKO over WT CD8$^+$ T cells were determined for each interaction anchor in three groups as in **b**, and Log2 fold changes were plotted in boxplot. In **b** and **c**, red line in the middle of box denotes median, box denotes interquartile range (IQR), and whiskers denote the most extreme data points that are no more than 1.5 × IQR from the edge of the box; the statistical significance was calculated using one-sided Mann-Whitney U test.

**d**. Workflow chart showing the analytical process of identifying chromatin loop hubs and assessing Tcf1 enrichment at loop anchors (See Methods for details).

**Shan and Li et al. Supplementary Figure 4**

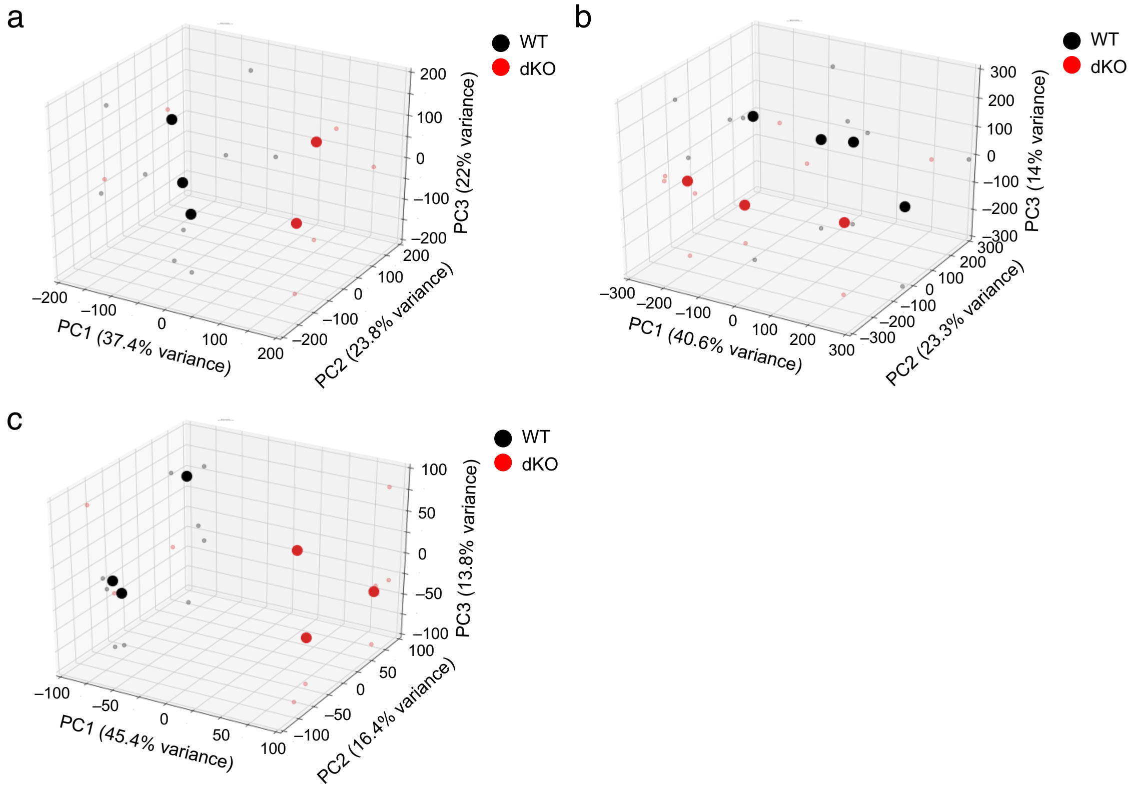


**Supplementary Figure 4. Multiomics characterization of WT and dKO CD8$^+$ T cells**.

WT and dKO CD8$^+$ T cells were isolated and subjected to DNase-seq (**a**), H3K27ac ChIP-seq (**b**), and RNA-seq (**c**) analyses. Shown are three-dimensional principal component analysis for multiple replicates in each assay. The small dots are projections of each replicate to respective 2D planes.

**Shan and Li et al. Supplementary Figure 5**

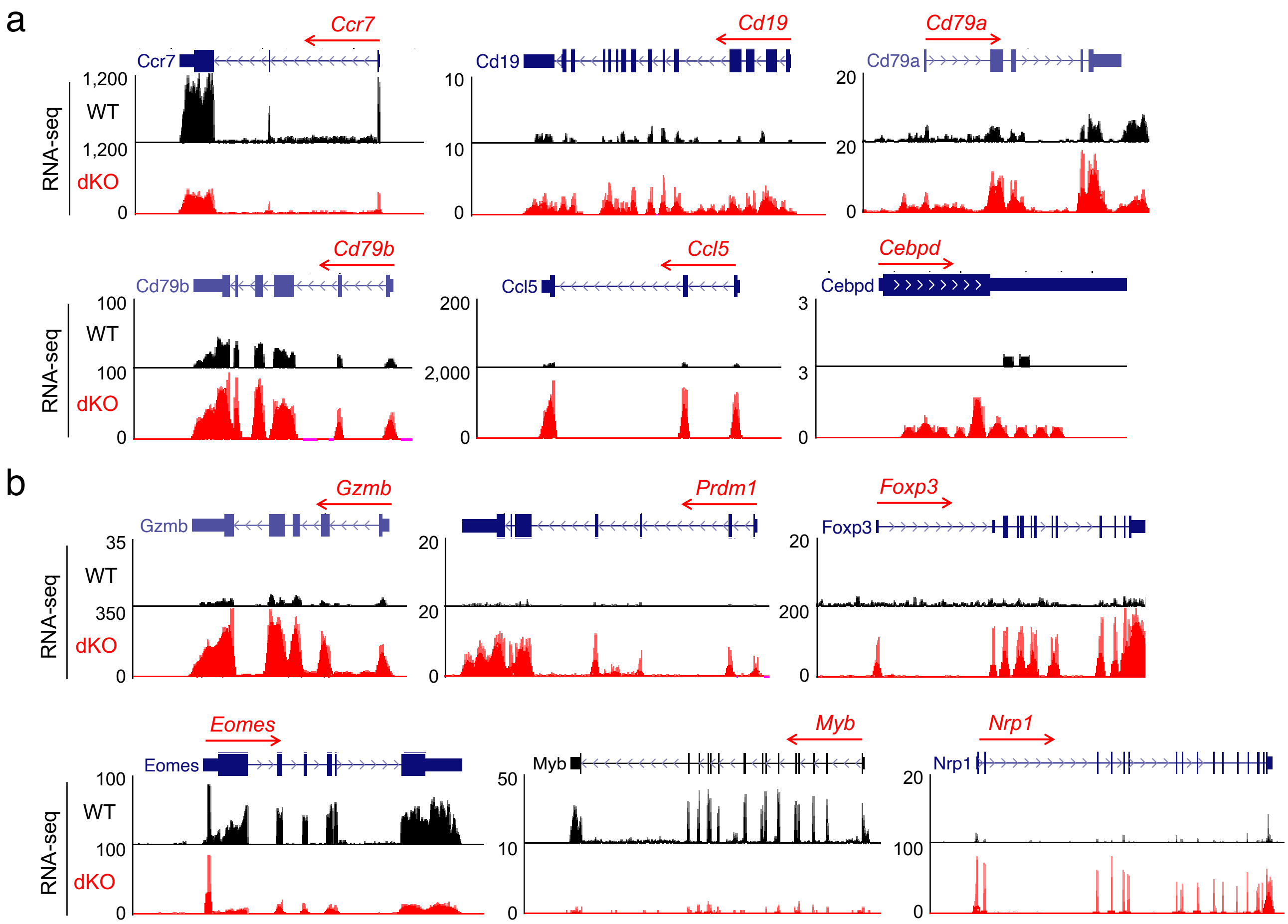


**Supplementary Figure 5. RNA-seq tracks at select gene loci**.

RNA-seq tracks are displayed on the UCSC genome browser for select differentially expressed genes between WT and dKO CD8$^+$ T cells. Note that *y*-axis values, which represent normalized FPKMs, are adjusted to visualize profound expression of changes in *Ccl5*, *Foxp3*, *Myb* and *Nrp1* genes.

**a**. Select T cell vs non-T cell lineage enriched genes.
**b**. Select effector CD8$^+$ T cell and non-CD8$^+$ T lineage enriched genes.

**Shan and Li et al. Supplementary Figure 6**

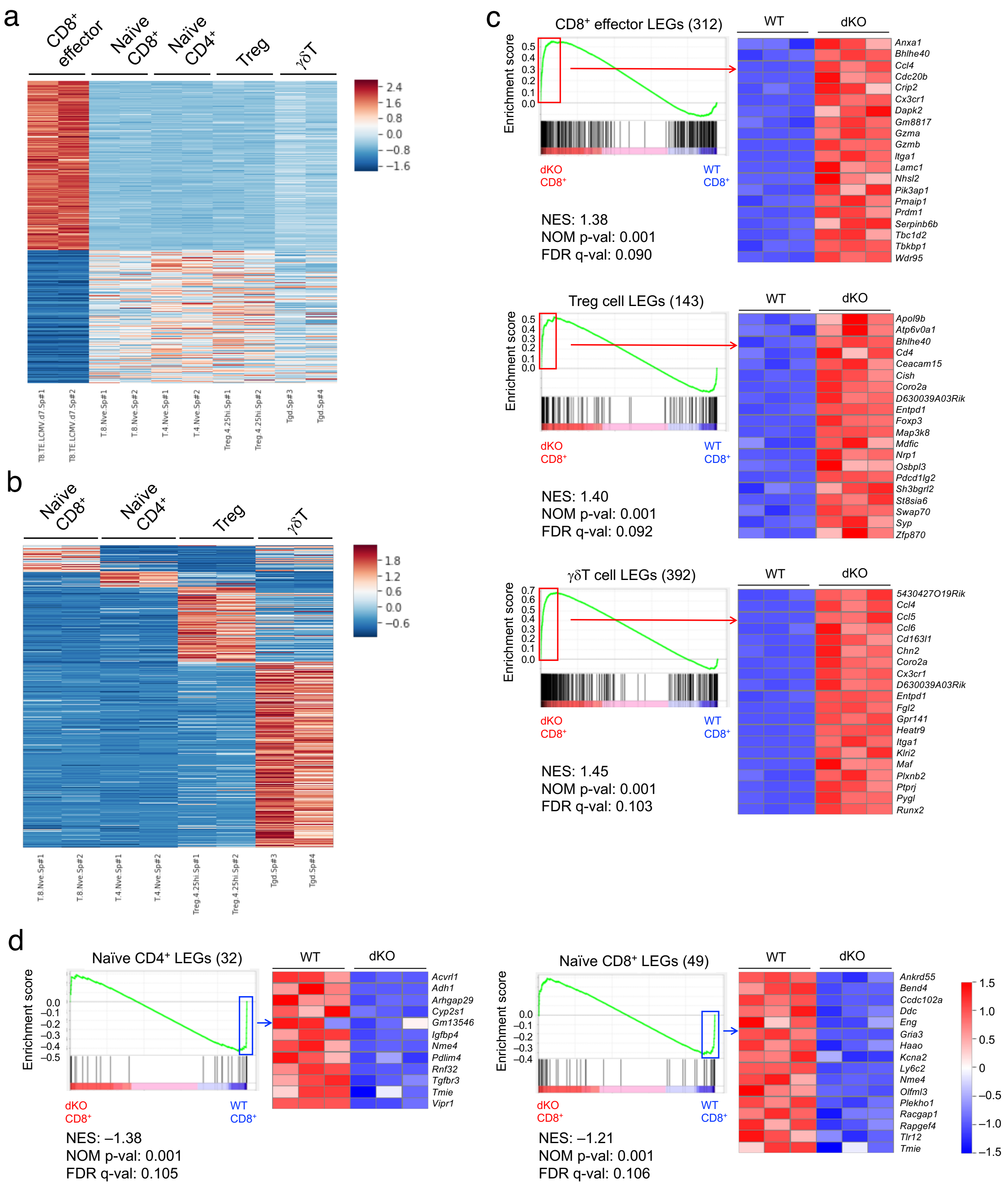


**Supplementary Figure 6. Tcf1/Lef1-deficient CD8$^+$ T cells were enriched for effector, Treg and γδT cell lineage-enriched gene (LEG) sets.**

**a**. Clustering analysis of T cell subsets identifies LEGs associated with effector CD8$^+$ T cells.

**b**. Clustering analysis of T cell subsets at naïve state identifies LEGs for naïve CD4$^+$, naïve CD8$^+$, Treg, and γδT cells.

**c**. Effector CD8$^+$, Treg, and γδT cell LEG gene sets are enriched in dKO over WT CD8$^+$ T cells, where 89 of 312 effector CD8$^+$ LEGs, 52 of 143 Treg LEGs, and 207 of 392 γδT LEGs genes are at the leading edge.

**d**. Naïve CD4$^+$ and naive CD8$^+$ T cell LEG gene sets are enriched in WT over dKO CD8$^+$ T cells, where 12 of 32 naïve CD4$^+$ LEGs and 16 of 49 naïve CD8$^+$ LEGs are at the leading edge.

For all GSEA, enrichment plot for each gene set is shown, and also marked are NES (normalized enrichment score), NOM p-val (nominal p values), and FDR q-val (false discovery rate q values) as output from GSEA. Red and blue rectangles mark genes in the leading edge, enriched in WT and dKO CD8$^+$ T cells, respectively. Top enriched genes in each set are shown in heatmaps, and all heatmaps use the same colour scale.

**Shan and Li et al. Supplementary Figure 7**

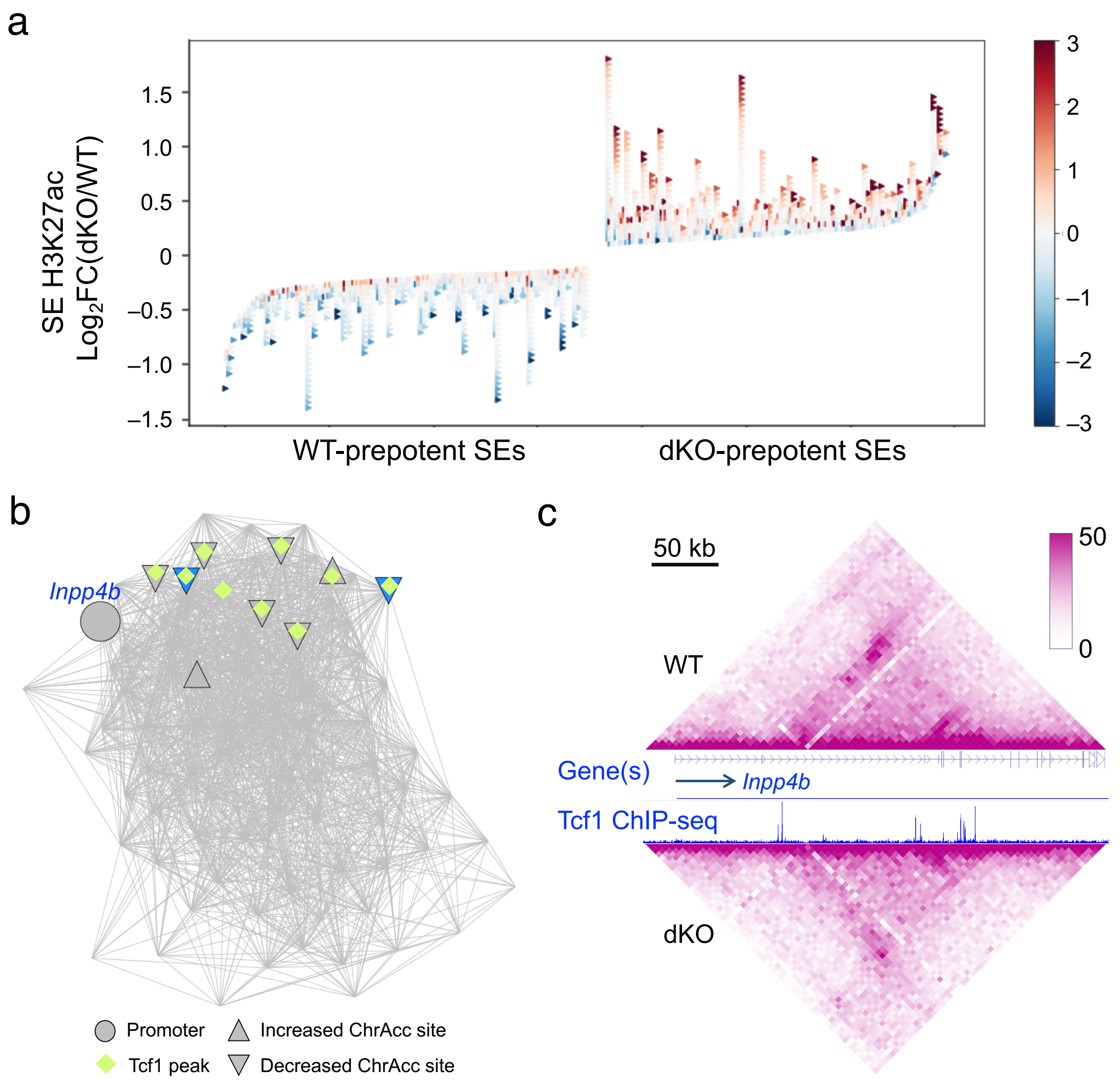


**Supplementary Figure 7. Tcf1/Lef1 TFs modulate super enhancer activity and chromatin interaction for target gene regulation.**

**a**. Stack heatmap showing correlation between H3K27ac changes in differential SEs and expression changes of SE-associated genes. Among WT- or dKO-prepotent SE groups, each column represents an SE, and all genes (marked as triangles) associated with the SE are stacked. The expression changes of all SE-associated genes (not limited to DEGs+DLEGs) are denoted with color code in the triangles, with the color scale shown on the right.

**b–c**. Tcf1/Lef1 TFs regulate *Inpp4b*, a T-cell lineage enriched gene, by promoting chromatin interactions. **b**. Network display of a WT-specific hub containing *Inpp4b*. The *Inpp4b* promoter is marked with a circle, and triangles filled with blue denote ChrAcc sites showing statistically significant decrease in dKO $CD8^+$ T cells. **c**. Two-dimensional display of changes in chromatin interaction within the *Inpp4b*-containing hub in WT and dKO $CD8^+$ T cells. Note that the one-dimensional tracks at the *Inpp4b* locus are in **Fig. 6e**, right panel.

**Shan and Li et al. Supplementary Figure 8**

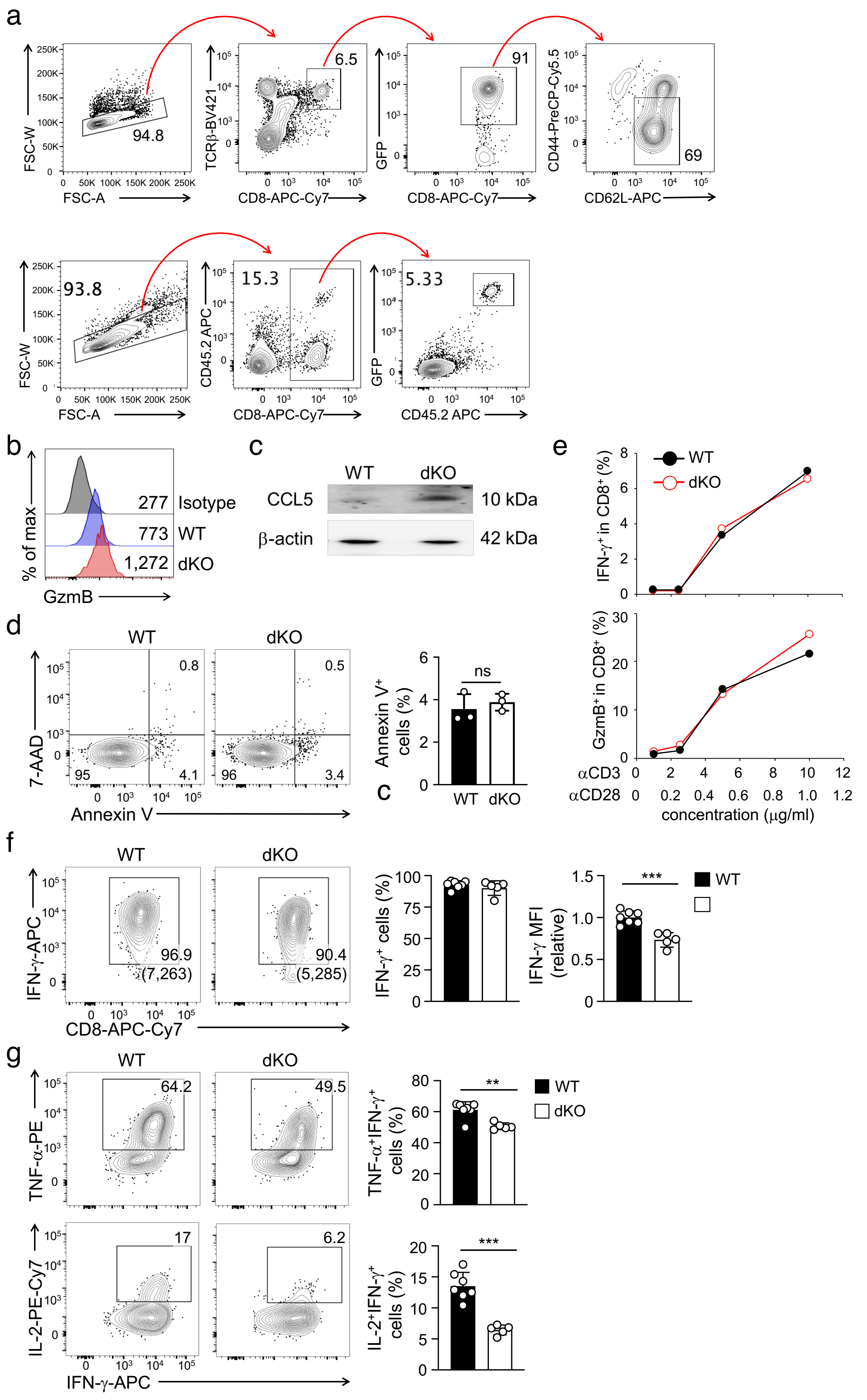

**Supplementary Figure 8. Tcf1/Lef1 TFs regulate differentiation of effector $CD8^+$ T cells**.

**a**. Gating strategies. The top row was used for analysis or sort-purification of naïve $CD8^+$ T cells for downstream analyses in **Fig. 9a–f**, **Supplementary Fig. 8b–d**. The bottom row was used for analysis of adoptively transferred and activated $CD8^+$ T cells in **Fig. 9h–k**, **Supplementary Fig. 8f–g**.

**b**. Validation of elevated granzyme B (GzmB) expression in naïve dKO $CD8^+$ T cells with intracellular staining, with gMFI marked.

**c**. Validation of elevated CCL5 production by naïve dKO $CD8^+$ T cells after 5-hr treatment *ex vivo* with PMA and ionomycin in the presence of Golgi Stop and Golgi Plug, by immunoblotting.

**d**. Detection of apoptotic cells in splenic $GFP^+CD8^+$ T cells from WT and dKO mice by Annexin V and 7-AAD staining. Cumulative data on the frequency of Annexin $V^+$ cells are means ± s.d. from two experiments.

**e**. Measurement of $CD8^+$ T cell activation threshold. WT and dKO $CD8^+$ T cells were isolated, and stimulated *ex vivo* with plate-bound anti-CD3 and soluble anti-CD28 at indicated titrating doses for 72 hrs, with Golgi Stop and Golgi Plug added during the last 5-hr incubation. IFN-γ (top) and granzyme B (bottom) were then detected and plotted against stimulation dosages. Data are average of two experiments.

**f**, **g**. Measurement of effector $CD8^+$ T cell functionality. On day 8 post-infection, splenocytes were stimulated with GP33 peptide for 5 hrs in the presence of Golgi Stop and Golgi Plug. Effector P14 T cells were detected as $CD45.2^+GFP^+CD8^+$ cells and intracellularly staining for IFN-γ (**f**), TNF and IL-2 (**g**). In representative contour plots in **f**, values denote percentages of IFN-$\gamma^+$ cells among effector P14 cells, and those in parentheses represent IFN-γ gMFI. In representative contour plots in **g**, values denote percentages of $TNF^+$ (top) or IL-$2^+$ cells (bottom) among effector P14 cells. Cumulative data on all these parameters are means ± s.d. from two experiments (right panels). Statistical significance in **d**, **f**, and **g** was determined with two-tailed Student's *t*-test. ns, not statistically significant; *, $p<0.05$; **, $p<0.01$; ***, $p<0.001$.
