# Supplementary material for: Tcf1 and Lef1 provide constant supervision to mature CD8+ T cell identity and function by organizing genomic architecture
Source: Nat Commun. 2021 Oct 6;12:5863. doi: 10.1038/s41467-021-26159-1 (PMC8494933; doi:10.1038/s41467-021-26159-1)
Supplement: Supplementary file 3 — Description of Additional Supplementary Files [file 41467_2021_26159_MOESM3_ESM.pdf]

Description of Additional Supplementary Files

File Name: Supplementary Data 1
Description: **Immune Cell Lineage Enriched Genes.** The Normalized Gene Table were downloaded from the Immunological Genome Project (http://www.immgen.org/) data browsers under entry GSE109125, and used to identify lineage-enriched genes for select immune cell types. Listed are lineage-enriched gene symbols in each cell type, together with its expression values for the cell type (in blue) along with all other cell types (in black) for direct comparison.

File Name: Supplementary Data 2
Description: **T Lineage Enriched Genes.** Transcriptomics data from the Normalized Gene Table (GSE109125) were used to identify lineage-enriched genes (LEGs) for each T cell subset. Listed are LEG symbols in each T cell subset, together with expression values for the subset (in blue) along with all other subsets (in black) for direct comparison.

File Name: Supplementary Data 3
Description: **Differentially Expressed Genes (DEGs) and Differential Lineage Enriched Genes (DLEGs) in Immune Cells.** The DEGs between WT and dKO $CD8^+$ T cells were threshold-based with criteria of ≥ 2-fold expression changes and adjusted p value <0.05. Expression values for all replicates are shown along with fold changes and adjusted p values. The DLEGs are derived from the leading edges based on GSEA, where gene symbols and corresponding LEG types are listed. The statistical significance is an output of CuffDiff.

File Name: Supplementary Data 4
Description: **Tcf1/Lef1 target genes linked to differential chromatin accessibility.** Differential ChrAcc sites between WT and dKO $CD8^+$ T cells that were linked to DEGs+DLEGs were clustered as in **Fig. 5a**. Listed for each ChrAcc cluster are genomic locations of the Diff ChrAcc sites, fold changes of ChrAcc, linked genes (gene symbols, expression changes and linkage rule), along with overlap with Tcf1 peaks (denoted with value of '1'). Note that one Diff ChrAcc site may be linked to more than one gene, and hence listed multiple times.

File Name: Supplementary Data 5
Description: **Tcf1/Lef1 target genes linked to super enhancers with differential activity.** Differential super enhancers (SEs) between WT and dKO $CD8^+$ T cells were identified and linked to DEGs+DLEGs. Listed for WT- or dKO-prepotent SEs are genomic locations of the SEs, fold changes in collective H3K27ac signal strength within SEs, linked genes (gene symbols and expression changes). Note that one SE may be linked to more than one gene, and hence listed multiple times.

File Name: Supplementary Data 6
Description: **Tcf1/Lef1 target genes linked to hubs with differential chromatin interactions.** Differential chromatin interaction hubs between WT and dKO $CD8^+$ T cells were identified and linked to promoters of DEGs+DLEGs within the hubs. Listed for WT- or dKO-specific hubs are genomic locations of the hubs, median of the fold changes of chromatin interaction scores, linked genes (gene symbols and expression changes). The statistical significance was estimated using one-sided Wilcoxon signed rank test. Note that one hub may be linked to more than one gene, and hence listed multiple times.
