# Supplementary material for: Tcf1 and Lef1 provide constant supervision to mature CD8+ T cell identity and function by organizing genomic architecture
Source: Nat Commun. 2021 Oct 6;12:5863. doi: 10.1038/s41467-021-26159-1 (PMC8494933; doi:10.1038/s41467-021-26159-1)
Supplement: Supplementary file 10 — Reporting Summary [file 41467_2021_26159_MOESM10_ESM.pdf]

nature research

Corresponding author(s): Hai-Hui Xue, Weiqun Peng

Last updated by author(s): Aug 9, 2021

Reporting Summary

Nature Research wishes to improve the reproducibility of the work that we publish. This form provides structure for consistency and transparency in reporting. For further information on Nature Research policies, see our Editorial Policies and the Editorial Policy Checklist.

Statistics

For all statistical analyses, confirm that the following items are present in the figure legend, table legend, main text, or Methods section.

n/a	Confirmed	
☐	☒	The exact sample size (*n*) for each experimental group/condition, given as a discrete number and unit of measurement
☐	☒	A statement on whether measurements were taken from distinct samples or whether the same sample was measured repeatedly
☐	☒	The statistical test(s) used AND whether they are one- or two-sided<br>*Only common tests should be described solely by name; describe more complex techniques in the Methods section.*
☐	☒	A description of all covariates tested
☐	☒	A description of any assumptions or corrections, such as tests of normality and adjustment for multiple comparisons
☐	☒	A full description of the statistical parameters including central tendency (e.g. means) or other basic estimates (e.g. regression coefficient) AND variation (e.g. standard deviation) or associated estimates of uncertainty (e.g. confidence intervals)
☐	☒	For null hypothesis testing, the test statistic (e.g. *F*, *t*, *r*) with confidence intervals, effect sizes, degrees of freedom and *P* value noted<br>*Give P values as exact values whenever suitable.*
☐	☒	For Bayesian analysis, information on the choice of priors and Markov chain Monte Carlo settings
☐	☒	For hierarchical and complex designs, identification of the appropriate level for tests and full reporting of outcomes
☐	☒	Estimates of effect sizes (e.g. Cohen's *d*, Pearson's *r*), indicating how they were calculated

Our web collection on statistics for biologists contains articles on many of the points above.

Software and code

Policy information about availability of computer code

Data collection	Sequencing is performed on Hi-Seq2000 of Illumina in single end read mode with read length of 50 bp (RNAseq), or in paired end read mode with read length of 150 nucleotides (ChIPseq, DNase-seq, and Hi-C). Flow cytometry data were collected on FACSVerse cytometer.
Data analysis	For nextgen sequencing data, the following was used: Juicer(v1.21.01), hiclib(v0.8.0 https://github.com/mirnylab/hiclib-legacy), Picard(http://broadinstitute.github.io/picard/), bowtie2(v2.2.5.), FastQC (v0.11.4), MACS2(v2.1.1), SICER(v1.1), edgeR(v.3.20.7.2), HOMER(v4.11), Cuffdiff (v2.2.1), Tophat(v2.1.0), GSEA(v4.1.0).<br>For HiC_hub analysis, please refer the algorithm at https://github.com/lux563624348/HiC_Hubs. Details were listed in the method section.<br>Flow cytometry data were analyzed on FlowJo v10.2 (TreeStar) and statistical analysis performed on Prism v8.0 (GraphPad).

For manuscripts utilizing custom algorithms or software that are central to the research but not yet described in published literature, software must be made available to editors and reviewers. We strongly encourage code deposition in a community repository (e.g. GitHub). See the Nature Research guidelines for submitting code & software for further information.

Data

Policy information about availability of data

All manuscripts must include a data availability statement. This statement should provide the following information, where applicable:

- Accession codes, unique identifiers, or web links for publicly available datasets
- A list of figures that have associated raw data
- A description of any restrictions on data availability

The data reported in this paper are tabulated in the Supplementary Materials and archived on the GEO database under accession number GSE164713.

Field-specific reporting

Please select the one below that is the best fit for your research. If you are not sure, read the appropriate sections before making your selection.

- [x] Life sciences
- [ ] Behavioural & social sciences
- [ ] Ecological, evolutionary & environmental sciences

For a reference copy of the document with all sections, see nature.com/documents/nr-reporting-summary-flat.pdf

Life sciences study design

All studies must disclose on these points even when the disclosure is negative.

Sample size	No sample size was predetermined through calculation. All multiomics data were collected from at least two independent experiments, in two-four replicates, which are sufficient to provide statistical power in nextgen sequencing data analysis.
Data exclusions	No data were excluded from analyses.
Replication	For all experiments, at least two biological replicates were analyzed in at least two independent experiments. All the replicates showed consistent reproducibility or distinct clusters in nextgen sequencing analyses.
Randomization	Mice are randomly selected from a pool of 6-12 weeks mice for phenotypic analysis or sorting for Hi-C or multiomics analyses.
Blinding	The investigators were not blinded to the group allocation during data collection or analysis. Blinding is not relevant for analysis of nextgen sequencing data.

Reporting for specific materials, systems and methods

We require information from authors about some types of materials, experimental systems and methods used in many studies. Here, indicate whether each material, system or method listed is relevant to your study. If you are not sure if a list item applies to your research, read the appropriate section before selecting a response.

Materials & experimental systems

n/a	Involved in the study	
[ ]	[x]	Antibodies
[x]	[ ]	Eukaryotic cell lines
[x]	[ ]	Palaeontology and archaeology
[ ]	[x]	Animals and other organisms
[x]	[ ]	Human research participants
[x]	[ ]	Clinical data
[x]	[ ]	Dual use research of concern

Methods

n/a	Involved in the study	
[ ]	[x]	ChIP-seq
[ ]	[x]	Flow cytometry
[x]	[ ]	MRI-based neuroimaging

Antibodies

Antibodies used	Antibodies used in this studies were listed in Method.
Validation	In house made rabbit anti-Tcf1 sera were validated by immunoblotting and immunoprecipitation (supporting data in Fig. S2a-S2c). The other antibodies are either validated by the manufacturers or in previous reports.

Animals and other organisms

Policy information about studies involving animals; ARRIVE guidelines recommended for reporting animal research

Laboratory animals	Laboratory animals used in this study are described in the methods section.
Wild animals	No wild animals were used in this study.
Field-collected samples	Study did not involve samples collected in the field.
Ethics oversight	All experiments are performed under the protocol approved by the Institutional Animal Use and Care Committees of the University of Iowa and Center for Discovery and Innovation, Hackensack University Medical Center.

Note that full information on the approval of the study protocol must also be provided in the manuscript.

ChIP-seq

Data deposition

- [x] Confirm that both raw and final processed data have been deposited in a public database such as GEO.
- [x] Confirm that you have deposited or provided access to graph files (e.g. BED files) for the called peaks.

Data access links<br>*May remain private before publication.*	Go to https://www.ncbi.nlm.nih.gov/geo/query/acc.cgi?acc=GSE164713<br>Enter token ijynsamollijfgr into the box
Files in database submission	GSM5016615 naive CD8 T cells, Tcf1 ChIPseq<br>GSM5016616 Tcf1-deficient CD8 T cells, Tcf1 ChIPseq<br>GSM5017016 WT naïve CD8, DNase seq, rep1<br>GSM5017017 WT naïve CD8, DNase seq, rep2<br>GSM5017018 WT naïve CD8, DNase seq, rep3<br>GSM5017019 Tcf1/Lef1-deficient naïve CD8, DNase seq, rep1<br>GSM5017020 Tcf1/Lef1-deficient naïve CD8, DNase seq, rep2<br>GSM5017661 WT naïve CD8+, HiC, Rep1<br>GSM5017662 WT naïve CD8+, HiC, Rep2<br>GSM5017663 Tcf1/Lef1-deficient naive CD8+, HiC, rep1<br>GSM5017664 Tcf1/Lef1-deficient naive CD8+, HiC, rep2<br>GSM5017665 WT naive CD8+, H3K27ac ChIP-Seq, rep1<br>GSM5017666 WT naive CD8+ input<br>GSM5017667 WT naive CD8+, H3K27ac ChIP-Seq, rep2<br>GSM5017668 WT naive CD8+, H3K27ac ChIP-Seq, rep3<br>GSM5017669 WT naive CD8+, H3K27ac ChIP-Seq, rep4<br>GSM5017670 Tcf1/Lef1-deficient naive CD8+, H3K27ac ChIP-Seq, rep1<br>GSM5017671 Tcf1/Lef1-deficient naive CD8+ input<br>GSM5017672 Tcf1/Lef1-deficient naive CD8+, H3K27ac ChIP-Seq, rep2<br>GSM5017673 Tcf1/Lef1-deficient naive CD8+, H3K27ac ChIP-Seq, rep3<br>GSM5017674 WT CD8 RNAseq rep1<br>GSM5017675 WT CD8 RNAseq rep2<br>GSM5017676 WT CD8 RNAseq rep3<br>GSM5017677 Tcf1/Lef1-deficient CD8 RNAseq rep1<br>GSM5017678 Tcf1/Lef1-deficient CD8 RNAseq rep2<br>GSM5017679 Tcf1/Lef1-deficient CD8 RNAseq rep3
Genome browser session<br>(e.g. UCSC)	https://genome.ucsc.edu/s/lux563624348/CD8%2DHP

Methodology

Replicates	One replicate is provided for Tcf1 ChIPseq. Three to four replicates are provided for H3K27ac ChIP-seq.
Sequencing depth	Tcf1 ChIPseq:<br>length of reads: 151 bps<br>paired-end<br>WT_na: raw reads, 54,855,805; unique reads: 33,543,448.<br>TKO_na: raw reads, 44,400,316; unique reads: 26,832,220.<br>H3K27ac:<br>length of reads: 151 bps<br>single-end:<br>WT_na: raw reads: 19,440,808, unique reads: 11,713709<br>dKO_na: raw reads: 26,762,606, unique reads: 12,235742<br>paired-end:<br>WT_na: raw reads: 33,397,929/44,728,983/42,672,064 unique reads: 27,494,246/34,958,043/34,733,452<br>dKO_na: raw reads: 32,837,708/31,664,726 unique reads: 25,716,725/ 28,642,749
Antibodies	Anti-Tcf1 antisera were made in-house by immunization of rabbits with full length Tcf1 recombinant protein. Anti-H3K27ac was purchased from Abcam (cat#: ab4729).
Peak calling parameters	Tcf1 peak calling by MACS2(v2.1.1): FDR < 0.05 , p<10(-5), Fold Enrichment >=4.<br>H3K27AC peak calling by SICER (v1.1): FDR < 0.01, windows size = 200bps and gap size = 400bps
Data quality	A total of 19,042 high confidence Tcf1 binding peaks were identified. For H3K27ac, a total of 65,563 and 82,213 H3K27ac peaks were identified in the pooled H3K27ac libraries for WT and dKO CD8+ T cells, respectively.

Software	MACS2(v2.1.1) and SICER (v1.1) were used for Tcf1 and H3K27AC peaking calling, respectively.

Flow Cytometry

Plots

Confirm that:

- [x] The axis labels state the marker and fluorochrome used (e.g. CD4-FITC).
- [x] The axis scales are clearly visible. Include numbers along axes only for bottom left plot of group (a 'group' is an analysis of identical markers).
- [x] All plots are contour plots with outliers or pseudocolor plots.
- [x] A numerical value for number of cells or percentage (with statistics) is provided.

Methodology

Sample preparation	Mouse spleens and lymph nodes were collected, and single cell suspension was made following standard procedures.
Instrument	FACSAria (BD Biosciences) for cell sorting, and FACSVerse (BD Biosciences) for analysis.
Software	FACSDiva (BD Biosciences) software was used for data collection. FlowJo v10.2 was used for data analysis.
Cell population abundance	≥95% on sorted cells, which was determined by flow cytometry analysis on post-sorted cells.
Gating strategy	Live lymphocytes were gated the known location through FSC-A/SSC-A. Doublets were removed by using FSC-A/FSC-W. CD4 and CD8 T cells cells were gated on TCRb+ cells.

- [x] Tick this box to confirm that a figure exemplifying the gating strategy is provided in the Supplementary Information.
